# Extended Reality—A Narrative Review of Current State and Best Use in Health Care Simulation

**DOI:** 10.1177/29941520251367055

**Published:** 2025-08-22

**Authors:** Jason Konzelmann, William B. Belk, Zaleha Abdullah Mahdy, Sindhu Bhargavi Mallala, Samantha Smeltzer, Mary Lee Carter

**Affiliations:** ^1^ Clinical Skills and Simulation Center, University of Central Florida – College of Medicine, Orlando, Florida, USA.; ^2^ Simulation and Innovative Education, Department of Clinical Education, Air Methods LLC, Longmont, Colorado, USA.; ^3^ Faculty of Medicine, Department of Obstetrics and Gynaecology, Universiti Kebangsaan Malaysia, Cheras, Malaysia.; ^4^ Lab for Immersive Technologies and Simulation, College of Public Health, George Mason University, Fairfax, Virginia, USA.; ^5^ Simulation and Integrated Simulation Operations, Orbis Education, Indianapolis, Indiana, USA.; ^6^ Learning Innovation, Center for Teaching and Learning, Parker University, Dallas, Texas, USA.

**Keywords:** augmented reality, virtual reality, mixed reality, extended reality, medical education, simulation-based education, educational technology

## Abstract

Extended reality (XR) is rapidly gaining ground in the field of health care simulation. Much remains to be explored in terms of how best to make use of this simulation modality while at the same time maintaining adherence to health care simulation standards of best practice. The effect of XR on learner cognitive load is controversial. On the contrary, XR is considered facilitative towards reducing cognitive load by enhancing audio-visual learning. However, technical adaptation requires adequate exposure to the device, in addition to which cybersickness can be a challenging side effect for users. Therefore, it is essential that prior to using XR simulations in health care education, both faculty implementers and learners should be provided with thorough training on how to use the technology effectively. Learning should be scaffolded by gradually increasing the complexity of XR simulations as learners become more proficient. Adapting simulation design principles for technology-enriched learning environments may help reduce cognitive load as well as positively affect performance. Novice learners benefit when they learn supporting information separately from complex procedural content. Extraneous cognitive load can also be minimized by avoiding redundant expressions of content. Future research should focus on improving XR-related hardware and software and assessing the effectiveness of XR as a simulation modality.

## Introduction

Extended reality (XR) technologies, including virtual reality (VR), augmented reality (AR), and mixed reality (MR), have revolutionized health care education by enhancing simulation-based learning environments.^1,2^ While classical simulation has long supported the bridging of theoretical knowledge and practical application, XR technologies further enhance this process through immersive, interactive, and realistic training experiences, using three-dimensional understanding facilitated through asynchronous learning platforms.^1^ However, the effective integration of XR into health care simulation remains a challenge, with differing approaches to components found in traditional simulation standards and best practices, including pre-briefing, observation, and debriefing.

This narrative literature review explores the current state of XR in health care simulation and aims to provide a comprehensive understanding of how best to utilize XR technologies to improve educational outcomes in health care settings. The review focuses on the following areas: the professional development needs of facilitators specifically related to the adherence to the Healthcare Standards of Best Practice (HSSOBP), position statement for designing XR simulations, technical standards for hardware and software, and cognitive load management strategies. For the purposes of this paper, the terms “health care education” and “health care simulation” should be construed to include all disciplines across medicine, nursing, and allied health.

## Mixed and Augmented Reality: Applications and Benefits in Medical Education

AR and MR have been described as disruptive technologies with potential uses in medical education.^2^ One review of the literature indicates as of early 2024, there were over 26,000 articles in medical and academic journals that include the terms AR, VR, and MR.^3^ Increases in computing power and interactive realism, combined with reduced costs of entry, have led to rapid advances in the implementation of applications, making these technologies less expensive than other available simulators.^1,4,5^ In addition, serious games and gamification of procedural skills teaching are becoming more commonplace, further driving AR and MR growth and demand.^4^ The Covid-19 pandemic also accelerated adoption of this technology.^6^

It has been noted that clinical surgical training, especially in the areas of surgery and anesthesia, is the most studied application of MR in medical education.^7^ Common areas of research include vascular access, particularly central line training, rachicentesis, endotracheal intubation, laparoscopy, and 3D assessment of anatomy.^1,7,4^ More recently, nonsurgical specialties such as emergency medicine, family medicine, gastroenterology, pediatrics, cardiology, radiology, and paramedicine have implemented MR for procedural training.^1,4^ In recent years, educators have found uses for AR and MR in interprofessional communication and social interactivity.^4^ Despite broader use, the translational benefits remain unclear, though some studies have attempted to consolidate the data.

Multiple studies were crystallized evaluating the efficacy and utility of XR in medical education.^5^Study outcomes were based on learner surveys evaluating confidence and satisfaction, technical examination scores between the control group and the study group, or success rate and speed of completion of the procedure. Overwhelmingly, it was found that XR study groups in this review performed better when compared to traditional teaching methods such as lectures and non-traditional teaching methods, including other forms of simulation-based education (SBE), and this is despite the observation that XR groups reported a higher overall cognitive load and significantly more side effects of XR usage, such as motion sickness and claustrophobia.^5^

Although weaknesses in the platform exist, the strengths likely outweigh them.^2,5^ Weaknesses include physiological reactions to the platform like motion sickness and nausea but were also identified in the visual field limitations, articulation with the real world, and technical limitations. Platform strengths are seen in areas of visualization, focused attention, spatial understanding of anatomical structure and function, and motivation to learn while also enhancing skill retention and confidence.^1–2,5^ It was concluded that, although the evidence is compelling that AR and MR improve learning outcomes, strong evidence is still lacking and that it is time to move from contemplating whether to use it, and instead focus on the how and when while also working to build evidence to demonstrate improved efficacy of learning.^2,5^ For a comparison of some of the benefits and challenges of using XR in health care simulation, see Table 1.

**Table 1.** Benefits and Challenges of Using XR in Health Care Simulation

Benefits	Challenges
Increased computer power progressively making XR less expensive than other simulators for long term use Procedural skills training enabled e.g., through serious games, for both surgical and non-surgical specialties Interprofessional communication and social interactivity learning are possible AI based comprehensive individual learner feedback and monitoring are possible Evidence shows that XR study groups perform better compared to traditional teaching methods	Physiological reactions e.g., motion sickness and nausea Visual field limitation Limited articulation with the real world Technical limitation e.g., cumbersome headsets Translational benefits remain unclear and are an area of future research

XR, extended reality.

AR and MR platforms are a promising adjunct to teaching and learning for medical education across all levels of providers, including paramedicine, nursing, and medicine. Furthermore, it can be broadly applied to diverse areas of medicine, including, but not limited to, anatomy and physiology, procedural training, surgical preparation, interdisciplinary learning, and pathophysiological presentations of illness. As computational and technological capabilities within the platform improve, the platform has become more accessible, which will broaden the available research base. While the growing body of evidence is supporting use of XR, particularly in areas of learner confidence and skills understanding, more research is needed in the areas of translational applications of learning from the simulation experience to the clinical space, with many universities designing their own XR cases and platforms.^4,7^

## Position Statement for Design of XR Simulations

Simulation-based Learning (SBL) is widely regarded as an optimal means of bridging the gap between theoretical knowledge and practical application.^8^ Integrating XR technologies (AR, VR, and MR) into SBL helps provide users with a multimodal experience, enabling an advanced level of realism, immersion, interaction, and higher performance.^9,10^ The absence of a pedagogical framework and comprehensive methodology may hinder the optimal use of XR technology.

**FIG. 1.**
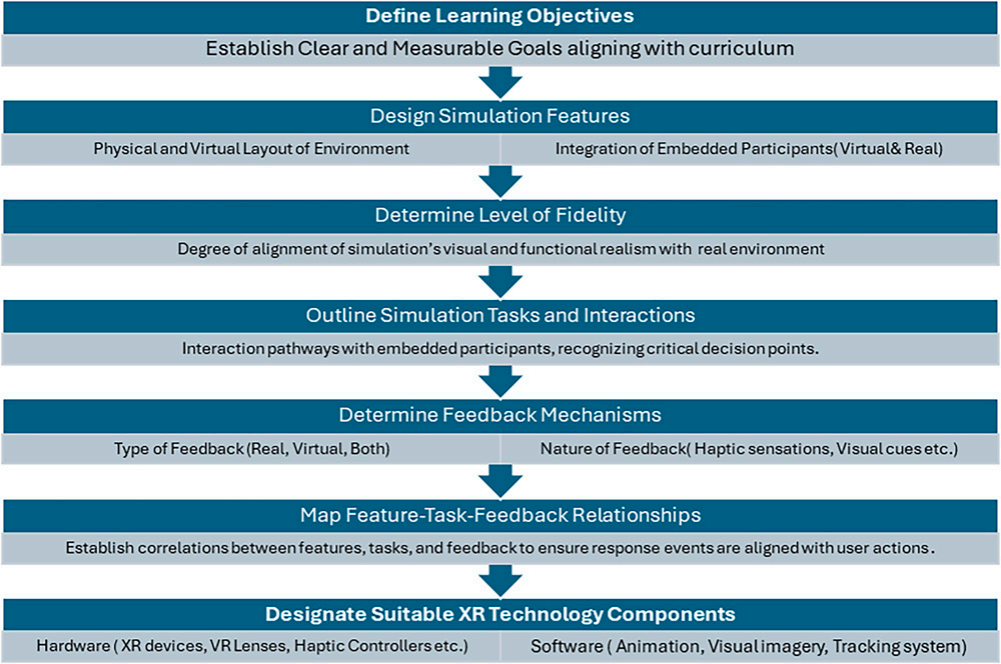
Flow for simulation-based learning design using XR technologies. XR, extended reality.

Ensuring alignment between simulation design and XR technology is crucial for creating a symbiotic relationship that supports and enhances immersion and multisensory interaction, fostering engagement and learning outcomes. Figure 1 outlines a suggested process of aligning XR technology components with learning goals for SBL experiences.

In 2021, a study was conducted in which researchers proposed a simulation design framework integrating components of simulation design and XR prototypes that can be adapted to a combination of simulation designs with different simulation modalities.^11^ The process of designing a simulation should commence with clearly defined learning goals, which will serve as a guide for the structure and design of the simulation. The subsequent aspect of delineating pertains to the simulation’s features, including the location and layout of the environment, the incorporation of embedded participants, and patient anamnesis. The accuracy and fidelity of the simulation rely on the degree of visual similarity between the simulation environment and the actual environment, which influences perceived realism and task performance.^12,13^

The next step is to define tasks within the simulation. This involves defining each task for learners, considering embedded participant interaction, potential challenges with each interaction, and embedded participant management associated with the learner.^11^

Defining the necessary feedback (real, virtual, or both) from the selected physical and virtual systems for the simulation is the final aspect in the simulation design process. This entails the customization of haptic feedback (such as pressure, vibration, and force), essential tactile sensations, holograms, and patient information displayed on the virtual monitor, which impact the user’s perception, psychological arousal, and interaction within the virtual environment.^14^ Establishing correlations between defined features, tasks, and feedback is vital for driving the simulation design based on triggers or events occurring in response to the user’s actions.^11^

Aligning the simulation design with the suitable XR technology improves the user experience, helps create a realistic multimodal training environment, and enhances real-time interactivity.^15,16^ This entails selecting the suitable hardware components, such as XR devices, VR lenses, HoloLens, and haptic controllers, as well as software elements, including animation, visual imagery, and tracking systems.

XR-based simulation does not adhere to a single pedagogical approach but rather spans a diverse spectrum of instructional designs. These range from instructor-led collaborative virtual environments, such as anatomy lessons conducted synchronously in shared virtual spaces, to autonomous, gamified modules where learners independently engage with clinical content and skills practice.^17,18^

While this narrative review does not attempt to categorize these modalities in detail, we acknowledge the significance of instructional design diversity and its alignment with different theoretical frameworks such as constructivism, cognitive load theory, and experiential learning. The choice of XR modality should be informed by the intended learning outcomes, the nature of the clinical task, and the learner’s level of experience. Future work is encouraged to explore and evaluate these varying pedagogical models in a more systematic and comparative manner.

## XR and Health Care Simulation Standards of Best Practice™

The growing use of XR technologies in health care simulation offers transformative potential, enhancing learner experiences and improving outcomes in clinical education. In addition, the effective use of XR technologies includes incorporating Healthcare Simulation Standards of Best Practice® (HSSOBP). Critical elements of these standards are needed in the realm of facilitation, prebriefing, and debriefing to ensure SBE meets the learner’s objectives and overall curriculum of the learners.^19,20^ Facilitators should be encouraged to engage in ongoing professional development through workshops, webinars, and conferences to keep pace with advancements in XR and its applications in health care simulation.

Facilitator development must extend beyond theoretical learning. Instructors should spend considerable time immersed in the virtual environments they plan to use for teaching. This practical experience is crucial for several reasons. First, it allows facilitators to fully understand the capabilities and limitations of XR technology, which is fundamental to designing effective, realistic simulations. By becoming proficient in the virtual environment, facilitators can ensure that their simulations are pedagogically sound and technologically feasible, thus ensuring meeting the HSSOBP facilitation standards.^17^ Understanding the scope of XR technology helps facilitators create simulations that are achievable and valuable, avoiding scenarios that may be too complex or beyond the capabilities of the technology.

Furthermore, familiarity with XR platforms enables facilitators to troubleshoot technical issues efficiently. Whether it is a technical glitch or a user error, facilitators who have significant experience navigating the virtual environment can resolve problems quickly, ensuring that training sessions proceed smoothly without interruptions. This technical competence is critical for maintaining the integrity of the learning experience, as extended interruptions due to technical difficulties can detract from the educational value of the simulation.^21^

Prebriefing, an essential component of simulation, plays a crucial role in setting the stage for successful learner engagement in the XR environment. It provides participants with the necessary context, learning objectives, and psychological safety, ensuring they are mentally and emotionally prepared for the simulation experience.^22,23^ Expanding on psychological safety, it is important for the learner to feel comfortable with the headset. Creating time for the learner to practice before the simulation and to be adequately prepared to engage meaningfully in the simulation ultimately enhances the effectiveness of the entire learning process.

One of the unique challenges presented by XR technologies in health care simulation is the debriefing process. Traditional simulation debriefing typically occurs in real time, with facilitators observing learners’ actions throughout the simulation. In XR-based simulations, however, debriefing may be asynchronous, meaning facilitators may not directly observe the simulation as it happens. Despite this difference, the goal of debriefing remains unchanged: to promote reflective learning that leads to improved clinical competence. The effectiveness of a simulation-based experience is closely tied to the quality of its reflective debriefing.^24^ In the case of XR simulations, facilitators must adapt their debriefing strategies to suit the asynchronous nature of the experience. This might include using recorded simulations, learner self-assessments, or structured reflective exercises to ensure that learners engage meaningfully with the content. Facilitators must be skilled in designing debriefing sessions that promote deep reflection and critical thinking, regardless of whether they are conducted in real time or asynchronously.

The integration of artificial intelligence (AI) into XR debriefing is an emerging area of interest. AI-driven systems have the potential to support asynchronous debriefing by analyzing learner performance data and generating tailored feedback.^21^ Natural language processing technologies are being developed to simulate facilitator dialogue and offer structured reflective prompts. These systems aim to replicate elements of traditional debriefing models, such as advocacy-inquiry, through conversational AI agents.

While this narrative review did not initially include a dedicated analysis of AI-enhanced debriefing due to the limited availability of peer-reviewed literature at the time of writing, we recognize the growing relevance of this topic. The use of AI in XR debriefing remains a rapidly evolving domain, and future research is needed to assess the pedagogical validity, learner perceptions, and ethical implications of these technologies. Although promising, current systems still lack the emotional nuance, adaptability, and contextual sensitivity of trained human facilitators^24^ The authors see this as an area of important future research.

The integration of XR technologies into health care simulation holds great promise, but its success hinges on the professional development of facilitators. Facilitators must be well-versed in both the technological aspects of XR and the educational principles that underpin effective simulation. This requires continuous professional development, including hands-on experience in virtual environments, technical troubleshooting skills, and an empathetic understanding of learner challenges. Furthermore, facilitators must adapt their debriefing strategies to accommodate the unique demands of XR simulations, ensuring that learners continue to benefit from reflective, educationally sound debriefing sessions. By adhering to these best practices, health care educators can leverage XR technology to enhance clinical education and improve patient care outcomes.

## Position Statement for Managing Cognitive Load in Extended Reality Simulation Implementation

Cognitive load refers to the amount of mental effort required to process information and perform tasks.^25^ It is influenced by the complexity of the task, the individual’s prior knowledge and experience, and the available cognitive resources.

Opinion and evidence regarding cognitive load in various XR modalities are mixed. XR can reduce cognitive load by enhancing audio-visual learning, allowing learners to focus on listening and viewing rather than reading and interpreting text.^26,27^ However, high engagement, informational overload, and unfamiliarity with virtual elements can lead to cognitive overload and acute stress.^28^ Evidence suggests that XR’s technical complexity and novelty may increase extraneous cognitive load, which is the unnecessary cognitive load imposed by instructional design or environmental factors. It has been found that AR glasses can unnecessarily increase cognitive load.^28^ In addition, adding task trainer-based problem completion exercises in VR significantly increased cognitive load and lowered performance, highlighting the need to control extraneous load when using new technology.^29^

Several measures can mitigate cognitive load in XR-based simulation (Table 2). Intrinsic load, which is the inherent difficulty of the task itself, can be managed by teaching supporting knowledge separately from procedural steps.^30,31^ Sequencing facts, concepts, and principles before procedural tasks can improve instructional efficiency. Extraneous cognitive load can be minimized by avoiding redundant content expressions. Learners perform better with multimedia lessons containing only animation and audio narration compared to those with redundant text.^32,33^ Overall, implementing XR in health care education requires strategies to manage cognitive load effectively, ensuring optimal learning outcomes and user experience.

**Table 2.** Measures to Optimize Learners’ Cognitive Load in Order to Facilitate Learning When Using XR as a Health Care Simulation Modality

Type of cognitive load	Measures to facilitate learning
Intrinsic	Teach supporting knowledge separately from procedural steps Break down complex tasks into smaller, more manageable steps Prioritize content relevance to align closely with curriculum and learning goals
Extrinsic	Avoid redundant content expressions Ensure intuitive and user-friendly XR environment Simplify user interfaces Offer thorough training to both faculty and learners on effective use of XR technology
Germane	Provide relevant feedback and guidance that support learning

XR, extended reality.

When designing XR simulations for health care education, it is important to consider the cognitive load imposed on learners. To manage intrinsic load, break down complex tasks into smaller, more manageable steps. Minimize extraneous load by ensuring that the XR environment is intuitive and user-friendly, thus avoiding unnecessary cognitive strain. Focus on optimizing germane load, which is the desirable cognitive load that facilitates learning and skills acquisition, by providing relevant feedback and guidance that supports learning. In addition, prioritize content relevance to align closely with curriculum and learning goals, simplify user interfaces to reduce cognitive overload, and offer thorough training to both faculty and learners on effectively using XR technology.

## Defining Technical Standards for XR Hardware and Software

Implementing XR into a health care curriculum requires adhering to technical standards to ensure the tools are effective, reliable, and safe.^32^ These standards encompass hardware specifications, software requirements, and product evaluation.

Hardware for XR includes head-mounted displays (HMDs), which can be tethered or untethered. Tethered HMDs, connected to external personal computers or consoles, offer higher processing power, superior graphics quality, and lower latency but are less mobile and have a complex setup. They can also be significantly more expensive, making scalability a consideration. Untethered HMDs provide greater mobility and ease of use, making them suitable for various training environments, though they may have lower processing power, higher latency, and limited battery life.^34^ Reliable high-speed internet access and product support are essential considerations, as some HMDs run applications using high-speed computers, while others operate locally or use cloud-stored content.^35^

When comparing XR software for health care education, it is necessary to consider applications, user interface, and support for moderated versus asynchronous learning environments.^36^ Software platforms range from custom-developed applications to third-party products. Key factors include the nature of immersive experiences (asynchronous or moderated), the ability to create and customize scenarios, content accessibility for users without headsets, and appropriate haptic feedback.^37^ In addition, the method of accessing content on the HMD, whether through direct download, computer connections, or mobile device management systems, is an important consideration.^38^

## Discussion

The integration of XR technologies in health care simulation represents a significant advancement in medical education, yet several critical areas warrant further investigation and development. This review has identified key themes that deserve particular attention from researchers and educators.

The translation of XR-based learning to clinical practice remains unclear. While studies consistently demonstrate improved learner confidence and technical understanding through XR simulation, there is limited evidence regarding the transfer of these skills to patient care settings. Future research should focus on measuring outcomes at Kirkpatrick levels 2–4, examining behavioral change, performance improvement, and ultimately, patient care outcomes. This aligns with the assertion that the field must move beyond questioning whether to use XR and focus instead on how and when to implement it most effectively.^2^

The management of cognitive load in XR-based simulation presents another area for investigation. Current evidence reveals a complex relationship between XR technology and cognitive burden. While XR can enhance audio-visual learning and reduce certain aspects of cognitive load, the technical complexity and novelty of these platforms may simultaneously increase extraneous cognitive load. Extraneous cognitive load varies according to the category of XR and the type of devices used. It is important to control or limit the extraneous cognitive load when using new technology. Nonetheless, it is pertinent to remember that unfamiliarity with virtual elements could expose students to cognitive overload and acute stress. Furthermore, research is needed to develop and validate specific strategies for managing cognitive load across different XR modalities and learning contexts.

The optimal application of XR across different types of clinical skills training requires additional study. Current evidence suggests that XR may be particularly effective for certain applications, such as procedural skills training and anatomical visualization, but its utility for developing soft skills and interprofessional communication remains less clear. Understanding where XR provides unique advantages over traditional simulation methods—whether in task training, manikin-based simulation, or standalone applications—would help guide more strategic implementation of these technologies.

Several limitations in current XR implementation must be acknowledged. The physiological effects of XR use, including motion sickness and disorientation, can significantly impact learner engagement and may limit the widespread adoption of these technologies. In addition, the variety of platforms and lack of standardization in what constitutes AR, MR, and VR creates challenges for both researchers and educators. The decision between developing institution-specific scenarios versus utilizing commercial products also presents ongoing challenges for educational programs.

The development of technical standards for XR hardware and software remains an evolving process. While untethered HMDs offer greater mobility and ease of use, they may sacrifice processing power and graphics quality. Establishing clear technical specifications that balance performance requirements with practical considerations like cost and scalability will be crucial for broader adoption in health care education.

While this review highlights the predominance of learner satisfaction and knowledge acquisition outcomes in XR simulation studies, we recognize the absence of higher-level evaluation metrics such as behavior change and patient outcomes. A structured analysis of these studies through the Kirkpatrick model would be valuable but lies beyond the scope of this narrative review. We encourage future systematic or scoping reviews to explore this dimension in depth. Finally, the professional development needs of simulation facilitators in the XR space require continued attention. The unique aspects of prebriefing and debriefing in XR environments, particularly in asynchronous learning situations, present new challenges for educators. Research into effective facilitator training programs and best practices for XR-specific debriefing methodologies would help ensure optimal learning outcomes.

As XR technologies continue to evolve and become more accessible, addressing these research priorities will be essential for maximizing their educational impact. Future studies should employ rigorous methodologies to evaluate the effectiveness of XR-based simulation across different health care disciplines and learning contexts, while also considering the practical challenges of implementation and the need for standardization in both technical and pedagogical approaches.

## Conclusion

As XR platforms are a promising adjunct to teaching and learning for medical education across all levels of providers, including paramedicine, nursing, and medicine. Furthermore, it can be broadly applied to diverse areas of medicine, including, but not limited to, anatomy and physiology, procedural training, surgical preparation, interdisciplinary learning, and pathophysiological presentations of illness. As computational and technological capabilities within the platform improve, the platform is becoming more affordable, which will have the effect of broadening the available research base. While the growing body of evidence supports the use of XR, particularly in areas of learner confidence and skills understanding, more research is needed in the areas of translational applications of learning from the simulation experience to the clinical space. Future research into the translational efficacy of XR simulation, especially at higher Kirkpatrick levels, as well as a deeper exploration into the diversity of pedagogical models and how these align with theoretical frameworks such as constructivism, cognitive load theory, and experiential learning, are important areas of inquiry. Furthermore, the integration of AI into debriefing processes presents a promising but underexplored area of research, particularly in supporting asynchronous reflective learning. By addressing these evolving needs, future research and implementation efforts can maximize XR’s potential to transform health care simulation and ultimately improve learner outcomes and patient care.

## Abbreviations Used

AI: artificial intelligence
AR: augmented reality
HMDs: head-mounted displays
HSSOBP: Healthcare Standards of Best Practice
MR: mixed reality
SBE: simulation-based education
SBL: simulation-based learning
VR: virtual reality
XR: extended reality
